# Understanding the impact of supervision on reducing medication risks: an interview study in long-term elderly care

**DOI:** 10.1186/s12913-017-2418-6

**Published:** 2017-07-06

**Authors:** J. A. Vermeulen, S. M. Kleefstra, E. M. Zijp, R. B. Kool

**Affiliations:** 1 Dutch Health Care Inspectorate (IGZ), Department Nursing and Long-Term Care, Utrecht, the Netherlands; 2 Dutch Health Care Inspectorate (IGZ), Department Risk Identification and Development, Utrecht, the Netherlands; 3 Radboud University Medical Center, Radboud Institute for Health Sciences, IQ Healthcare, Nijmegen, the Netherlands; 4 Dutch Health Care Inspectorate (IGZ), PO Box 2518, 6401 DA Heerlen, the Netherlands

**Keywords:** Medication safety risks, Medication incident reports, Safety culture, Nursing homes, Elderly, Supervision

## Abstract

**Background:**

In 2009, the Dutch Health Care Inspectorate (IGZ) observed several serious risks to safety involving medication within elderly care facilities. However, by 2011, high risks had been reduced in almost all the organisations we visited. And yet the IGZ analysed too the alarming increase in the number of incidents arising in the self-reported national indicator of medication safety between 2009 and 2010. The aim of this study was to understand the factors that can explain this contradiction between the increase in self-reported medication incidents and the observation of the IGZ in reducing the risks to medication safety through supervision.

**Methods:**

We interviewed health care professionals of ten care facilities, visited by the IGZ, who were involved in, or responsible for, the improvement of medication safety in their institutions. As outcome measures we used the rate of medication safety risk per facility; the perceptions of the participant with regard to the reports of medication incidents; the level of medication safety of the facility; the measures used to improve medication safety; and the supervision of medication safety.

This was a mixed methods study, qualitative in that we used semi-structured interviews, and quantitative, by calculating risks for the different organisations we visited. The findings from both study methods resulted in a comprehensive view and an in-depth understanding of this contradiction.

**Results:**

The contradiction between the increase in self-reported medication incidents and the observation of reduced risks was explained by three themes: activities designed to improve medication safety, the reporting of medication incidents, and, lastly, the impact of supervision. The focus of the IGZ on issues of medication safety stimulated most elderly care facilities to reduce medication risks. Also, a change in the culture of reporting incidents caused an increase in the number of reported incidents.

**Conclusions:**

Supervision contributed to an improvement in actions geared towards reducing the risks associated with the safety of medication. It also increased a willingness to report such incidents. The more incidents reported are therefore not necessarily a sign of an increase in the risks, but can also be considered as a sign of a safer culture.

**Electronic supplementary material:**

The online version of this article (doi:10.1186/s12913-017-2418-6) contains supplementary material, which is available to authorized users.

## Background

Medication incidents are a major risk to public health [1]. A secure means of supplying and taking the medication, such as its safe and appropriate supply and administration, seems to be a challenge all over the world. This is especially true for nursing homes and homes for older people. In Norway, one third of nursing home residents experienced at least one medication error a year [2]. In the United Kingdom, this figure was even as high as two out of three [3].

There are several reasons why the safety of medication is such a challenge in the care of older people. The first problem is polypharmacy, that is the use of five or more medicines. This is reported for 30 to 45% of the older people. This increases to ten or more medicines for 20% of the people aged over 75. Research suggests that among people living in Dutch residential care for older people 40 % is being over-treated in a sense that too much medication is prescribed while some medicines are not or no longer necessary. Avoidable side effects and adverse interactions between medicines are common risks for people who are prescribed multiple medications [4]. Secondly, several medicines are not suitable for older people because of the patient’s reduced cognition or metabolic capacity and functioning in many systems, such as the liver, brain, heart or muscles. Thirdly, co-morbidity increases the risk of less effective pharmaceutical treatment [5].

Errors take place during different stages of the medication process, from prescribing, preparation and delivery, to actually taking pills, and the administration involved surrounding the whole process of medication [6]^**.**^ There are several policies to reduce medication errors in nursing homes and homes for the elderly [7]. For instance, changing the behaviour of qualified health care professionals is crucial. This can be achieved in part by recording agreements, adhering to guidelines and protocols, and through clear communication [8]. Furthermore, adequate introduction of technology, such as systems for the automated dispensing of medication or electronic prescribing, play an important role in improving the processes of medication [9].

The Dutch Health Care Inspectorate (IGZ, see Table 1) has, since 2009, chosen medication safety as one of the special topics for its supervision of long-term care for older people. This is both because of the major risks and the opportunities to prevent them. Between May 2009 and March 2010 the IGZ visited 93 nursing homes and homes for older people in order to identify possible medication safety risks. It concluded that significant improvements were essential. There were several reasons for this view. There was insufficient consultation between organisations and pharmacists or general practitioners. Health care professionals, in general, lacked the skills and expertise in relation to medication safety. Finally, many organisations had no safe medication distribution systems [10].

**Table 1:** The Dutch Health Care Inspectorate (IGZ)

The Dutch Health Care Inspectorate (IGZ) is an agency of the Ministry of Health, Welfare and Sport. It is the official regulatory body charged with supervising the quality and safety of healthcare services, prevention activities and medical products in the Netherlands. The IGZ has organised its supervision in several ways in order to ensure compliance with professional standards and guidelines and to ensure patient safety. The most important methods are supervision based around incidents and analyses of various types of risk information, also known as risk-based supervision.
If risks are identified, then the IGZ visits a nursing home or a home for older people. This method of supervision consists of general supervision and, what is defined as thematic supervision. In this case the IGZ conducts visits to several organisations based on a specific theme common to the whole sector. This action is the result of research or incidents such as medication safety. The IGZ then assesses one or more risks depending on the diversity and severity of the risks identified within a specific organisation. This assessment is based on reviewing documents relating to the quality management systems, such as care protocols and patient files, but also to reviewing the communications with, for example, care managers, care professionals and patients.
The organisation must then take measures to improve safety and thus reduce the risks. These improvements and their results must then be reported to the IGZ. The IGZ will then conduct a follow-up visit to assess the implementation of the improvement measures. The IGZ is mandated to use enforcement measures if the organisation does not comply and there is insufficient faith in the organisation to realise the improvements in time. For instance, the IGZ can impose intensified supervision of an organisation. This may involve frequent visits, announced or unannounced, and consultations with the board. They can involve the organisation as a whole or just one of its departments. The IGZ can also penalise the organisation including prohibiting it, temporarily or even permanently from accepting new patients.

**Fig. 1:**
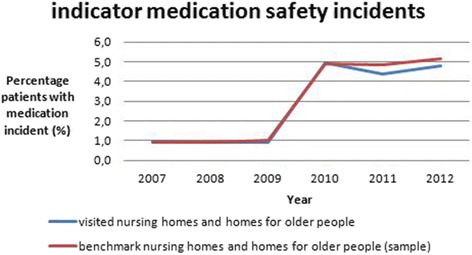
Percentage of patients that encountered a medication incident in 1 month

During follow-up visits in 2011, the IGZ observed that within a year such high risks had been reduced by almost all the organisations where the IGZ had identified them during its previous visits [11]. The IGZ now attempted to confirm this observation through an analysis of the national indicator of medication safety incidents over the years 2007-2012 (see Additional files 1 and 2). The indicator represents the percentage of patients that encountered a medication incident during the month prior to the measurement of the indicator. All Dutch nursing homes and homes for older people are obliged to report this indicator once a year. The indicator is self-reported and the benchmarked outcomes are reported to all organisations after correcting for their casemix. The national indicator of the organisations visited, as well as a benchmark group, showed an alarming increase of medication safety incidents between 2009 and 2010 as shown in Fig. 1.

This increase seems to contradict the results of the supervision activities and the conclusions of the thematic supervision by the IGZ. One would expect fewer medication incidents because of the improvements observed in medication safety in the elderly care facilities visited. We aim in this paper to understand the paradox posed by the increase in the reports of medication incidents at the same time as reduction in the risks observed. In addition, we aim to create insight into whether monitoring the national indicator of medication safety incidents is an efficient way to identify the impact of supervision by regulatory bodies such as the IGZ.

## Methods

### Design

Firstly, we looked at the organisations visited by the IGZ inspectors within the context of the supervision of medication safety between 2009 and 2011. We then collected all their risk calculations. Secondly, we conducted semi-structured interviews by telephone with the professionals who were involved in, or responsible for, the improvement of medication safety in the nursing homes and homes for older people which we selected.

### Data collection risk calculations

The homes for older people were visited in 2010. During the visits, two inspectors supervised 11 medication themes that may cause risks. These themes were: the transfer, ordering, monitoring, surveillance, availability, storage, preparation and dispensing of medication; the training and competencies of health care professionals; the quality system; and the handling of medication errors. There was also supervision of additional themes in nursing homes if deemed applicable. These themes were the guidelines for the prescription of medication and the presence of small pharmacies located within the care facilities but without a pharmacist.

In order to assess the level of medication safety, the IGZ collected information by reviewing documents relating to the quality management systems, looked at patient files and spoke with employees. All the themes were scored separately for ‘risk’ or ‘no risk’. The IGZ presented the results in a final report following each visit.

If risks were identified, then the organisation had to take measures to improve safety which had to be reported to the IGZ. Moreover, the IGZ conducted a follow-up visit a year later to assess the implementation of the improvement measures. These results were also presented in a report.

The number of medication safety risks observed during the visits was contained in these final reports in 2010 and 2011. The authors collected the risks from these reports and present them in Table 3.

### The setting and interviews of the participants

We made a subjective selection of ten of the 93 elderly care facilities which were visited by the IGZ in 2009-2010 and during the follow-up. We selected organisations which reported the highest increase in medication incidents per inhabitant in 2011 compared to a year before and the highest decrease in reports of medication incidents. We used subjective sampling to ensure diversity in experiences with this risk indicator. We approached the organisations by letter. Afterwards, we called the organisations to arrange an appointment for an interview by telephone. Before starting the interview, we informed the interviewees a second time about what to expect, their rights and obligations, and explained that they were still allowed to refuse to participate. One care facility declined to participate because of a lack of time and a second care facility was already represented by another location of the same organisation. Therefore, two other organisations were invited to participate from the same categories as the ones which did not want to participate. Finally, the sample consisted of ten care facilities for older people. There was no relationship between the researchers and the care organisations. The number of organisations participating would be enlarged if the results showed variation. Saturation was reached after ten interviews.

### Data collection interviews

We used a semi-structured interview guide (Table 2) [12]. We performed a literature search for medication safety and added questions related to supervision activities in order to develop this interview guide.

**Table 2:** Semi-structured interview guide

Main topics	Examples of questions
Medication incident reports	What is the cause of the increase or decrease in the number of medication incident reports within the organisation? What could explain the national trend showing an increasing number of reports of medication incidents?
Medication safety level	What were the main conclusions with regard to medication safety during the visit of the IGZ in 2010? What is the current medication safety level within the organisation?
Safety improvement measures	Which measures for improving medication safety were taken by the organisation in recent years and why?
Supervision	What was the effect of the IGZ’s supervision on the medication safety level within the organisation? Did collaboration with other institutions or organisations contribute to the improvement in medication safety and why, or why not?

One researcher (JAV) interviewed all participants in October and November 2013. The interviews lasted from between 15 to 30 min. They were all audio taped and transcribed verbatim. We performed a member check, that is we presented the audiotapes to the interviewees in order to correct factual inaccuracies and allow the participants to reflect and comment on the accuracy and validity of the information. Eventually, all interviews were included in its original form.

The level of risk found in all the organisations was calculated by analysing the reports from the IGZ inspectors.

### Analysis of the interviews

The interviews were transcribed and analysed using the principles of Grounded Theory, a qualitative research method focusing on the identification of concepts that emerge from study interviews or observation [13]. Two researchers coded the transcripts to minimise subjectivity. The researchers used ATLAS.ti 7 to facilitate the coding process. Coding is the interpretative process in which conceptual labels are given to the data [13]. Two researchers (JAV and SMK) independently analysed the content of the first three transcripts and, drawing conclusions from their observations, identified initial codes (open coding). We merged codes again into unique codes and a framework for these codes was developed for further use (axial coding). JAV analysed the rest of the transcripts. New codes could then be added to the coding framework. Saturation was reached after ten interviews. Codes that related to the same phenomenon were grouped into categories and finally themes were identified. JAV and SMK discussed categories and themes and consensus was reached between both researchers. A third, and external, researcher (RBK) was consulted in order to review the codes, categories and themes. The COREQ (consolidated criteria for reporting qualitative research) checklist was used for reporting the results (see Additional file 3) [14].

## Results

### Quantitative results

Risks were identified in 2010 in 45% ((52/115)*100) of all the items assessed. This is shown in Table 3. The individual results from the ten care facilities which were visited showed a large variation in the risks observed. The themes most frequently identified as a risk were: the monitoring and storage of medication; the training and competencies of health care professionals; and the handling of medication errors (see Additional file 4). In 2011, the rate of risks observed decreased by almost 30% (45% -((20/115)*100)).

**Table 3:** The number of medication safety risks observed during the visits of the IGZ to the ten elderly care facilities

		2010		2011	
Organisation	Total potential risk themes	Risk	No risk	Risk	No risk
1	11	4	7	0	11
2	11	3	8	0	11
3	11	5	6	5	6
4	11	4	7	0	11
5^a, b^	13	0	13	-	-
6^a^	12	11	1	8	4
7	11	8	3	2	9
8	11	7	4	0	11
9^c^	11	4	7	-	-
10^a^	13	6	7	1	12
Total	115	52	63	20	95

^a^nursing homes; up to two extra themes were supervised

^b^no risks, so follow-up visits were not needed

^c^improvement activities were demonstrated to the IGZ, and so, a follow-up visit did not take place

### Qualitative results

#### Participants

Table 4 shows the demographic characteristics of the interviewees.

**Table 4:** Demographic characteristics of the participants

Respondent	Sex	Profession	Involved in visits? Yes/No
1	F	Care Manager	Y^a^
2	F	Pharmacist	Y
3	F	Policy advisor	N
4	M	Care Manager	N
5	F	Policy maker	Y^a^
6	F	Nurse	Y^a^
7	F	Location manager	Y
8	F	Nurse	Y
9	F	Medical director	Y
10	M	Medical director	N

^a^The participants were not present during the visits by the IGZ. However, the participants received feedback after the visit and were responsible for taking safety improvement measures and/or reporting the improvement activities and results to the IGZ

### Analysis

The qualitative analysis of the interviews resulted in eight categories from which three themes emerged: activities designed to improve medication safety; the reporting of medication incidents; and the impact of supervision (Table 5).

**Table 5:** Themes, categories, codes and quotes to understand the contradiction of the increase of medication incident reports and the reduction in medication safety risks

Theme	Category	Codes	Representive Quotes
I. Activities designed to improve medication safety	Improvements made based on the IGZ visits	Reducing emergency supply of medication; agreement with pharmacy; constructing medication working groups; agreements managing and administering medication; double-checks; training; ‘do not disturb’ logo; electronic prescribing; transition to another pharmacy; reformulating protocols	‘We formed a medication working group, with a board member as chairman, with different kinds of care professionals.’ (interviewee 10, care manager) ‘The agreements with the pharmacists on monitoring medication safety and both their responsibility, and that of the organisation itself were reformulated over time.’ (interviewee 10, care manager) We received medication from several community-based pharmacies. We were not at all happy with that. In the end, we went along with one party.’ (interviewee 3, medical director)
	Improvements made based on the organisation taking its own initiative	Internal audit; form a commission for incident reports; consultation of pharmacist and general practitioner; agreement with pharmacy; (reformulating) guideline reporting incidents; constructing medication working groups; electronic prescribing; ‘do not disturb’ logo; regular training; double-checks	‘One of our focus points was to introduce the double check on medication and ‘do not disturb’ logos during distribution.’ (interviewee 8, policy maker) ‘We use coloured jackets with a ‘do not disturb’ logo while distributing medication. However, psycho geriatric patients may not understand the meaning of the jackets, and sometimes ignored them, which can be difficult.’ (interviewee 5, location manager)
	Improvements made through collaboration with third parties	Referring to guidelines and protocols; agreement with pharmacy; consultation of another care facility/location, professional association, knowledge centre, general practitioner, pharmacy	‘Furthermore, we consulted pharmacists and reported on, for example, prescribing behaviour. Based on this information, we reviewed critically protocols for distribution.’ (interviewee 3, medical director)
II. Reporting of medication incidents	The internal causes of increased reports	Willingness; safe culture; clear what to report; automatic behaviour; no fear; lower threshold	‘It has become automatic. Employees do not have to worry that they might get punished if they report an incident. Previously, that feeling prevailed. That implies we had to reduce the fear of reporting and to make it clear that it is just an instrument to improve quality.’ (interviewee 7, care manager) ‘The barrier to report incidents was reduced. We saw an abrupt increase in reported errors. But if improvements are achieved and the quality and safety of medication is guaranteed, then we will notice that it actually contributes to reducing errors.’ (interviewee 4, policy advisor)
	The internal causes of fewer reports	Automated medication dispensing system; simplified processes; raised awareness; training; ‘do not disturb’ logo; managing medication per patient; verbal report	‘We organized training for professionals, to, among other things, stimulate their awareness about medication safety.’ (interviewee 1, medical director) ‘A lot of medication was forgotten, and was not administered or prepared well. By using the automated medication dispensing system, quite a lot of these errors were prevented.’ (interviewee 6, nurse)
	The national causes of increased reports	Raised awareness; small incidents are reported; digitisation of reporting; higher complexity of processes; workload; more complexity of care; taboo -subject is broken	‘The amount of medication incident reports used to be very low. But we could not say that nothing was wrong. We especially concluded: employees did not report errors. We realised that we had to stimulate that they would report.’ (interviewee 10, care manager)
III. Impact of supervision	The effects of the IGZ visits	Focus on details; speeds improvement processes; stimulates collaboration; raises awareness; objective judgment; catalyst; makes funding available	‘The visits of the Health Care Inspectorate causes alarm bells to ring.’ (interviewee 4, policy advisor) ‘It accelerates the process and made our medication process very accurate.’ (interviewee 1, medical director) ‘Also it matters to the board whether the Health Care Inspectorate indicates improvement is necessary. It opens doors or makes investments possible. Therefore, as professionals, we sometimes ‘used’ the Inspectorates’ authority to make necessary changes which we weren’t able to establish ourselves, unfortunately.’ (interviewee 2, pharmacist, responsible for medication care in nursing homes)
	The lack of effect of the IGZ visits	Intrinsic motivation; critical employees; good internal working environment	‘Honestly, I think that most of the improvements came from ourselves. We wanted things to get better, so we took care that things were getting better.’ (interviewee 3, medical director) ‘Did the Inspectorate have any influence on the willingness to report incidents? I don’t think so. It is merely an internal development, concerning how to deal with each other, what is the management style, how do employees experience reporting? So I think it is above all an internal matter. I don’t think the visits or the report of the IGZ contributed to that change of culture.’ (interviewee 10, care manager)

### Theme 1: Activities designed to improve medication safety

This theme is divided into three categories: (1) the improvements made based on the IGZ visits; (2) the improvements made based on the organisation taking its own initiative; (3) the improvements made through collaboration with third parties.

### Improvements made based on the IGZ visits

Nursing homes and homes for older people reported having made several improvements based on the conclusions of the first visit of the IGZ. Frequently recurring themes during the interviews were: the investment in employee training in medication safety; the introduction of coloured jackets with a ‘do not disturb’ logo worn by care professionals during their distribution and dispensing of medication; and the introduction of double - checks during the distribution and dispensing of medication which was not packed by an automated medication dispensing system. Medication working groups were also established, who were responsible too for pharmacotherapeutic and pharmacy consultations. Finally, small pharmacies located within care facilities but without a pharmacist were closed down.

### Improvements made based on the organisation taking its own initiative

Care facilities also made agreements with pharmacies and achieved a transition to automated medication dispensing systems and electronic prescribing. Furthermore, most of the interviewees emphasised that they had started to organise routine internal audits. Based on these audits, they started to review medication safety protocols.

### Improvements made through collaboration with third parties

Care facilities used the internal audit data in a variety of ways. Firstly, for achieving improvements in medication safety, secondly for external audits or accreditations and, finally, for external reporting to, for example, health insurance companies and the IGZ. These audits often took place in collaboration with pharmacies and hospital pharmacies. In addition, organisations asked research institutes to support them in improving medication safety by means of staff training, risk- and improvement management and calamity evaluation. Care facilities referred to professional standards and guidelines in order to review their medication safety protocols.

### Theme 2: The reporting of medication incidents

The three categories related to this theme which emerged were: (1) the internal causes of increased reports; (2) the internal causes of fewer reports; (3) the national causes of increased reports.

### The internal causes of increased reports

All the interviewees agreed that the focus on medication safety caused a change in the culture of reporting. This focus was not only achieved by the visits of the IGZ but also by the attention given by patient organisations and health insurers. Health care professionals started to realise that reported incidents were not used for punishment, but rather to provide input for improvements.

### The internal causes of fewer reports

All interviewees shared the opinion that many errors during the preparation, distribution and dispensing of medication were prevented. The simplification of the preparation, distribution and dispensing process was considered to be crucial. For example, care facilities which showed a decrease in the registration of medication incident reports said that the main cause of the decline was the automation of the medication process by introducing an automated medication dispensing system.

### The national causes of increased reports

As mentioned before, the increased attention to medication safety has increased an awareness of the relevance of analysing incidents and improving care processes in order to prevent incidents from occurring. The interviewees believed that this increased the willingness to report and consequently the number of medication incidents reported. It was also was noted that the growing complexity of health care and its processes could be responsible for the increased number of medication incidents reported.

### Theme 3: The impact of supervision

Two clear categories were identified within this theme: (1) the effects of the IGZ visits; (2) the lack of effect of the IGZ visits.

### The effects of the IGZ visits

According to most of the interviewees the specific supervision of medication safety, in addition to the regular supervision, had certainly contributed to the awareness and improvement in medication safety. Care facilities considered the IGZ as an objective and independent organisation which had the knowledge and authority to identify areas of care in which a minimum number of conditions were required to ensure a high quality. They view the IGZ was functioning here as a catalyst and was thus able to speed up improvements, the institutions considered this supervision as an important boost to their own activities for improving the quality of care.

### The lack of effect of the IGZ visits

Not all interviewees attributed the improvements to the activities of the Inspectorate. The interviewees mentioned that a positive working environment affected the intrinsic motivation of health care professionals. This motivation stimulated them to think critically and thereby to be able to improve their working practices.

## Discussion

We aimed, in this study, to understand the paradox posed by the increase in medication incident reports at the same time as the reduction in observed risks. Consequently, we aimed to provide insights into whether monitoring the national indicator of medication safety incidents is an efficient way to identify the impact of supervision.

However, several implications of the supervision by the IGZ regarding medication safety in elderly care facilities can be identified. First of all, it suggests that the increase in incidents reported to the national indicator of medication safety was merely an expression of an increased willingness to report, instead of an expression of the increased medication risks. From this perspective, the contradiction between the improvements in medication safety by supervision and the increased number of medication incident reports, as mentioned in the introduction, seems to be logical. The willingness to report might be considered as a sign of a safer culture with regard to medication. However, even though the trend analyses of the number of incidents showed an alarming increase, the IGZ should better be cautious in using this national indicator as a sign of the impact of supervision of medication safety. After all, the high number of incidents reported was not necessarily an indicator of increased risks. The reactions of the interviewees suggest that it is in fact the other way around in the sense that the fewer incidents reported, the higher the risks of medication errors. Former research has confirmed this phenomenon. A study into two Australian hospitals by Westbrook et al. [15] showed that the hospital with the higher number of incident reports experienced a lower actual rate of prescribing errors. The researchers concluded that the higher the number of medication incidents means a lower risk to the patient [15].

This study also shows the risks of using self-reported indicators. Several studies have confirmed that self-reporting can undermine the validity and reliability of data [16]. This could be explained in part by the small percentage of incidents occurring which the health care professionals actually report [15]. However, even though self-reported indicators, such as the number of medication incidents, may be unsuitable for indicating the impact of supervision, these indicators can certainly identify possible areas for improving safety. Yet, a health care inspectorate needs indicators with a high predictive value for unsafe or irresponsible care if it is to be able to explore the impact of its supervision [17]. For this purpose, a quantified measure could be developed, based on reliable and valid data [16].

Thirdly, this study suggests that the thematic supervision by the IGZ has contributed to a reduction in the medication risks among the level of health care providers. Thematic supervision of medication safety resulted in several quality improvement activities, a review of protocols and a change in culture of reporting incidents. Health care professionals started to realise that reporting incidents is not about punishing and blaming. It is, instead, essential for improving the safety of care. Most care facilities considered this supervision as a useful driving force for improving care safety. Using the advice of an authority such as the IGZ, meant that most nursing homes and homes for older people felt stimulated to organise improvement activities and to reduce the risk of medication incidents.

### Strengths and limitations

A limitation of this study is that the relationship between supervision activities and improvements is hard to understand. This requires insight into, mostly, complicated external factors. Van Dishoeck et al. [18] designed a theoretical chain of impact which could offer insight into this process. It explained that supervision activities can have an affect not just on health plans but also on the media, citizens, politicians and care unions. For their part, these stakeholders have an influence upon the compliance of health care professionals and organisations with the recommendations for improving care which follow from supervision [19]. Also another study on the impact of supervision indicated that improved adherence to guidelines is only partly attributable to a supervision programme. This programme, based on a guideline for midwives to counsel pregnant women to give up smoking, helped to improve counseling by making midwives aware of it and giving them an extrinsic motivation to provide such counseling. However, adherence to guidelines depended on several other factors too. The most important are motivation and professional and environmental factors. The impact of supervision on the quality and safety of health care might be mostly indirect and limited [18, 20].

Another limitation is that the two researchers were IGZ- employees. This might have caused social desirability bias, that is the tendency of the participants to answer the questions in a favourable manner. Secondly, this might have caused confirmation bias, that is the tendency to interpret the information from the interviews in a way that confirms the hypotheses of the IGZ researchers. We tried to limit the impact of these biases by the participation of the third researcher who was an external one and who supervised the analysis. Furthermore, we stressed in the introduction to the interviews that the interviewer was not an inspector with daily supervision tasks but their role was to work as a researcher for the IGZ. Finally, the participants were told that the results would be presented anonymously.

A third limitation is that the researchers have not achieved the stage of theoretical sampling. The selection was based on data gathered previously by the IGZ. Therefore, the researchers obtained maximum variation in the organisations participating by selecting those which reported an extreme increase or decrease in the number of reports of medication safety and by, as far as possible, selecting participants who were involved in the IGZ visits. By an appropriate subjective selection, saturation was reached after ten interviews.

A final limitation is that only a small number of organisations offering long-term elderly care were visited. Therefore, it is hard to draw general conclusions about these results for all Dutch elderly care facilities. However, despite the small sample of organisations selected, we ensured the quality of the research through a variety of means, such as by using different researchers to ensure internal validity, by conducting a member check of the interviews and by using the COREQ checklist. Nevertheless, when focusing on evaluating the impact of supervision, future research should draw on a larger sample of organisations in order to quantify the results and draw general conclusions.

However, the mixed method character of this study is one of its strengths. In addition to the analysis of quantitative data such as the national indicator of medication safety incidents and the risk calculations by the IGZ, interviews contributed to gaining a better understanding of the impact of supervision. This helped to explain the paradox posed by the increase in the number of medication incident reports. Furthermore, saturation was reached after ten interviews as almost all interviewees agreed on the impact of supervision [21].

### Implications and future research

The key finding for daily practice of supervision is to be aware of the risk of using self-reported indicators to assess the effects of supervision. In the Netherlands, as in many countries, many quality indicators are collected by organisations themselves. Future research could also use indicators based on, for example, administrative data to explore whether the same conclusions could be drawn.

Secondly, the interviewees stated that the visits of the IGZ worked as a boost or an inducement for actual compliance. It would be interesting to explore whether and how these visits of the IGZ contribute to actual compliance. For instance, exploring the difference in the impact on compliance between an advisory and a supervisory attitude of the inspector during the visit. It would, furthermore, be interesting to explore, in future research, whether a visit of this external catalyst has more impact on improving quality as opposed to performing quality improvements driven by ‘intrinsic motivation’ in organisations which are not visited.

Finally, the lessons learned from this study could also be useful for other Dutch supervisory bodies, or health care inspectorates outside the Netherlands. However, the differences in supervisory practice should always be taken into account.

## Conclusion

The interviewees indicated that supervision focused on medication safety, attributed to a transformation in the policy toward the medication safety of nursing homes and homes for older people. Most elderly care facilities and their professionals working for them stated that the IGZ was a useful catalyst and a driving force for reducing medication risks. This view was supported by a major decline in risks assessed by the IGZ. They also indicated that the culture of reporting had changed resulting in more self- reported medication incidents. Interpreting self-reported indicators in order to assess the impact of supervision should therefore be carried out with caution. An increased number of reported incidents is not necessarily an indicator of a higher level of risk.

## Additional files

Additional file 1: Indicator medication safety incidents year 2007_2008_2009. This file contains self-reported indicators from all care facilities over the years 2007-2009. The indicator represents the percentage of patients that encountered a medication incident during the month prior to the measurement of the indicator. (XLSX 49 kb)

Additional file 2: Indicator medication safety incidents year 2010_2011_2012. This file contains self-reported indicators from all care facilities over the years 2010-2012. The indicator represents the percentage of patients that encountered a medication incident during the month prior to the measurement of the indicator. (XLSX 36 kb)

Additional file 3: Research checklist COREQ. This file contains a 32-item checklist with consolidated criteria for reporting qualitative studies. (DOC 67 kb)

Additional file 4: The number of medication safety risks 2010-2011. This file contains the individual results from the ten care facilities; the number of medication safety risks observed during the visits of the IGZ. (XLSX 18 kb)

## Abbreviations

COREQ: Consolidated criteria for reporting qualitative research
IGZ: Dutch Health Care Inspectorate
